# Palindrome-Mediated Translocations in Humans: A New Mechanistic Model for Gross Chromosomal Rearrangements

**DOI:** 10.3389/fgene.2016.00125

**Published:** 2016-07-12

**Authors:** Hidehito Inagaki, Takema Kato, Makiko Tsutsumi, Yuya Ouchi, Tamae Ohye, Hiroki Kurahashi

**Affiliations:** ^1^Division of Molecular Genetics, Institute for Comprehensive Medical Science, Fujita Health UniversityToyoake, Japan; ^2^Genome and Transcriptome Analysis Center, Fujita Health UniversityToyoake, Japan; ^3^Department of Molecular Laboratory Medicine, Faculty of Medical Technology, School of Health Science, Fujita Health UniversityToyoake, Japan

**Keywords:** palindrome, inverted repeat, cruciform, chromosomal translocation, gross chromosomal rearrangement

## Abstract

Palindromic DNA sequences, which can form secondary structures, are widely distributed in the human genome. Although the nature of the secondary structure—single-stranded “hairpin” or double-stranded “cruciform”—has been extensively investigated *in vitro*, the existence of such unusual non-B DNA *in vivo* remains controversial. Here, we review palindrome-mediated gross chromosomal rearrangements possibly induced by non-B DNA in humans. Recent advances in next-generation sequencing have not yet overcome the difficulty of palindromic sequence analysis. However, a dozen palindromic AT-rich repeat (PATRR) sequences have been identified at the breakpoints of recurrent or non-recurrent chromosomal translocations in humans. The breakages always occur at the center of the palindrome. Analyses of polymorphisms within the palindromes indicate that the symmetry and length of the palindrome affect the frequency of the *de novo* occurrence of these palindrome-mediated translocations, suggesting the involvement of non-B DNA. Indeed, experiments using a plasmid-based model system showed that the formation of non-B DNA is likely the key to palindrome-mediated genomic rearrangements. Some evidence implies a new mechanism that cruciform DNAs may come close together first in nucleus and illegitimately joined. Analysis of PATRR-mediated translocations in humans will provide further understanding of gross chromosomal rearrangements in many organisms.

## Introduction

DNA palindromes consist of two units of identical sequences connected in an inverted position with respect to each other. In palindromes, the sequences on the complementary strands read the same in either direction. In other words, the complementary sequence appears in the same strand in an inverted orientation. Palindromic DNA can consequently form specific tertiary structures, namely, single-stranded “hairpin” or double-stranded “cruciform” DNA. Such unusual DNA tertiary structures are called non-B DNA structures (Sinden, 1994; Wang and Vasquez, 2014). These non-B DNA structures are presumed to be generated in a cell under specific situations, although their *in vivo* existence is still a controversial subject.

Hairpin structures can be formed when the double helix DNA is dissociated into single-stranded DNA molecules at the palindrome. Such single-stranded DNA might occur during DNA or RNA synthesis during replication or transcription. On the other hand, cruciform formation starts from unwinding of the center of the double-stranded palindromic DNA, followed by extrusion at the center of the palindrome to form an intra-strand base-paring of each strand. As the DNA unwinds, the cruciform gets bigger. Cruciform formation requires an under-twisted state, that is, negative superhelicity, of the DNA. Such unusual DNA structure itself could have an impact on DNA replication, repair, transcription, or other important biological pathways (Inagaki and Kurahashi, 2013). The DNA regions that potentially form non-B DNA structures often manifest genomic instability that induces gross chromosomal rearrangements (Pearson et al., 2005; Tanaka et al., 2005; Maizels, 2006; Raghavan and Lieber, 2006; Mirkin, 2007; McMurray, 2010).

## Palindrome-mediated chromosomal translocations in human sperm

The best-studied palindromic sequences are the breakpoint sequences of the constitutional t(11;22)(q23;q11.2) translocation, a well-known recurrent non-Robertsonian translocation in humans. Balanced carriers are healthy but often have reproductive problems such as infertility, recurrent pregnancy loss, and offspring with Emanuel syndrome (Carter et al., 2009; Ohye et al., 2014; Emanuel et al., 2015). Breakpoint analysis of 11q23 and 22q11 revealed that these regions contain a large palindrome of hundreds of base pairs that is extremely AT-rich (Kurahashi et al., 2000a, 2007; Edelmann et al., 2001; Kurahashi and Emanuel, 2001a; Tapia-Páez et al., 2001). These so-called palindromic AT-rich repeats (PATRRs) have been identified at both breakpoints on chromosomes 11 and 22 and are named PATRR11 and PATRR22, respectively. These PATRRs have several features in common. Both are several hundred base pairs in length and have greater than 90% AT content. They manifest nearly perfect palindromes without spacer regions but share little homology between the two chromosomes.

The most prominent feature of the t(11;22) translocation is that *de novo* translocations frequently arise at a similar breakpoint location. Translocation-specific PCR with primers flanking the breakpoints on chromosomes 11 and 22 can detect all of the t(11;22) junction sequence in the translocation carriers (Kurahashi et al., 2000b). We performed PCR at the single-molecule detection level using sperm DNA from normal healthy men with the 46, XY karyotype as template. Some DNA aliquots tested positive for t(11;22)-specific PCR products while others were negative, suggesting that the PCR detected *de novo* t(11;22) translocations (Kurahashi and Emanuel, 2001b). The frequency was about one in 10,000. However, when the DNA of blood cells or cheek swab cells from the same men was analyzed, no translocation could be found. Furthermore, all of the lymphoblastoid cell lines or cultured fibroblasts examined also tested negative in PCR analysis. These results imply that the t(11;22) translocation arises in a sperm-specific fashion. There is no evidence for the occurrence of the t(11;22) translocation during female gametogenesis because of the limited availability of human oocytes for testing. However, in *de novo* t(11;22) families, analysis of the parental origin of the translocation chromosomes using the polymorphic feature of PATRR11 and PATRR22 revealed that all of the *de novo* t(11;22) translocations were of paternal origin, supporting a hypothesized sperm-specific mechanism of t(11;22) translocation formation (Ohye et al., 2010).

## DNA secondary structure in the palindrome: hairpin or cruciform

What is behind the sperm-specific occurrence of the PATRR-mediated translocation? It is not unreasonable to discuss the mechanism leading to the t(11;22) translocation in the context of DNA secondary structure. The DNA secondary structure at the PATRR is potentially evidenced by the fact that a polymorphism within the PATRR affects the *de novo* t(11;22) translocation frequency (Kato et al., 2006; Tong et al., 2010). PATRR11 and PATRR22 have size polymorphisms in the general population due to deletion within the palindromic region. Carriers with long symmetric alleles preferably produce *de novo* t(11;22) translocations more frequently than carriers with PATRR asymmetric arms. These data indirectly but strongly implicate the presence of DNA secondary structure during translocation formation.

One hypothesis to explain the sperm specificity of the t(11;22) translocation is that it develops during DNA replication. Sperm production involves many cell divisions, each requiring DNA replication. During DNA replication, single-stranded DNA is generated in the template DNA for the synthesis of not only the lagging strand DNA, but also the leading strand (Azeroglu et al., 2014). When the replication fork comes to the palindromic region, a long single-stranded DNA is formed, inducing the formation of a single-stranded hairpin structure. The stalling of the replication fork produces DNA breakage at the palindromic region that can potentially induce translocations.

Because the germ stem cells in men replicate about 23 times per year, mature sperm from older men have undergone a greater number of replication cycles. The frequency of *de novo* point mutations in sperm cells increases according to the age of the sample donor (Crow, 2000; O'Roak et al., 2012). If the t(11;22) translocation is mediated by replication, the frequency of the *de novo* t(11;22) translocation should be higher in sperm from older men than in younger men for a similar reason. A previous analysis of the t(11;22) translocation suggested, however, that there is no tendency for an increase in t(11;22) translocation frequency in the sperm of older men (Kato et al., 2007).

**Figure 1:**
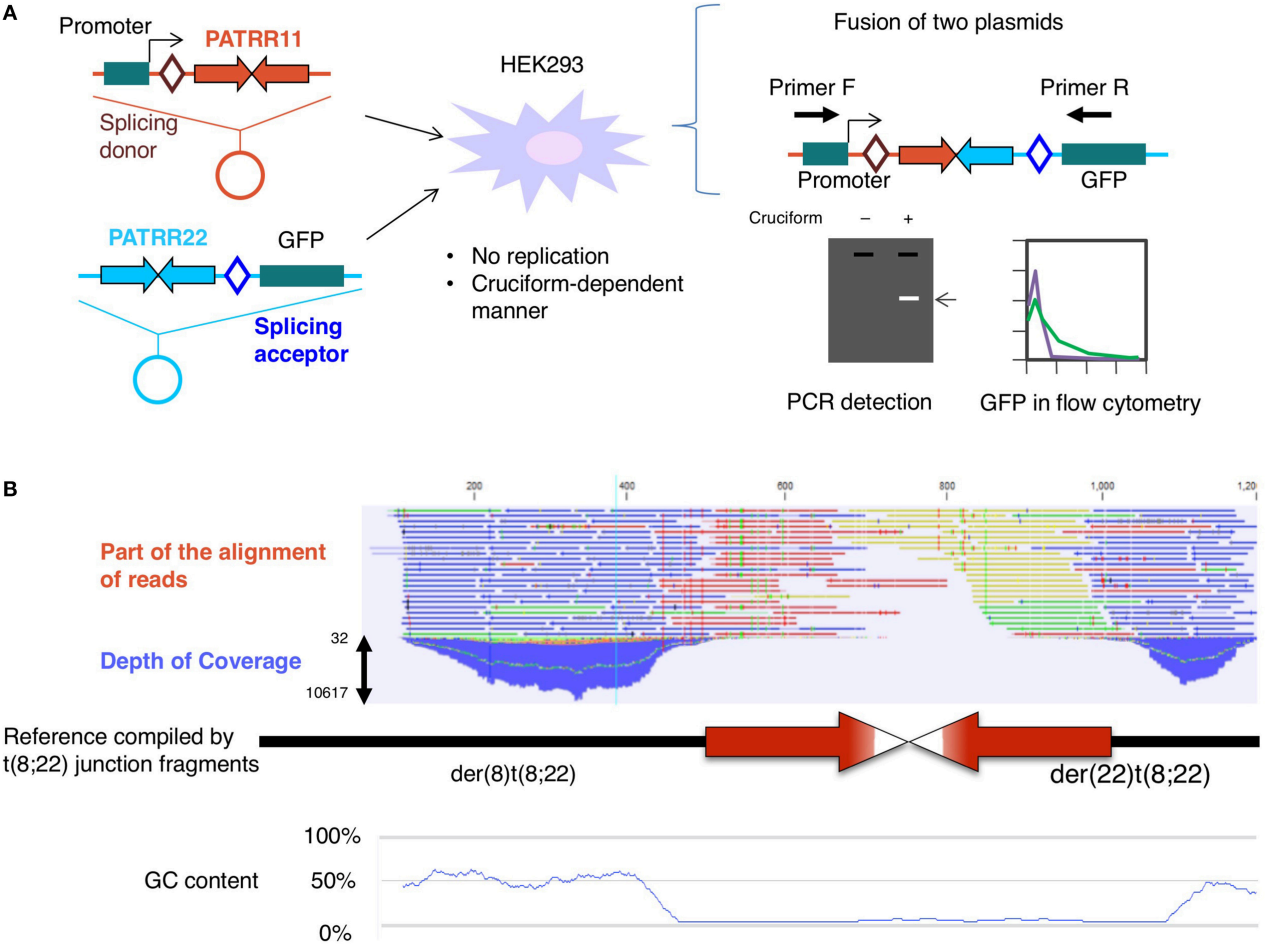
**(A)** Translocation model system. Two plasmids, one harboring a promoter, splicing donor, and PATRR11, and the other carrying PATRR22, a splicing acceptor, and a coding region of the GFP gene, were simultaneously transfected into HEK293 cells. After 24 h, fusion molecules generated by joining of the PATRR11 and PATRR22 at the center were detected by PCR or GFP-positive cells were monitored by flow cytometry (Inagaki et al., 2013). **(B)** Determination of the PATRR8 sequence by next-generation sequencing. Although the depth of the coverage was low at the center of the palindrome, massive parallel sequencing was able to fill the entire region of the palindrome (Mishra et al., 2014).

To determine the involvement of DNA replication in translocation formation, we established a model system for the t(11;22) translocation in cultured cells by using plasmids harboring PATRR11 or PATRR22 (Inagaki et al., 2009). Both plasmids were transfected into the HEK293 human cell line and we monitored the fusion of the different plasmids at each PATRR using GFP expression or translocation-specific PCR (Figure 1A). The results indicated that a translocation-like reaction took place. In this reaction, both PATRRs were cleaved at the center of the palindrome and joined via non-homologous end-joining in a similar manner to the human t(11;22) translocation. Crucially, the plasmids had no replication origin for human cells, which means that the translocation took place without DNA replication.

## Post-meiosis hypothesis for PATRR-mediated translocations

On the other hand, it is possible that the translocation is mediated by another secondary structure, the DNA cruciform. In our model system, the plasmids were purified from *Escherichia coli* using a standard alkaline lysis method. Plasmid DNA isolated from *E. coli* has a strong negative superhelicity. If the plasmid has a palindromic region, the negative superhelicity facilitates cruciform extrusion (Kurahashi et al., 2004). Under an alkaline condition that induces denaturation of the plasmid DNA during purification, most of the PATRR-harboring plasmids extrude cruciform structures. Via the use of a non-denaturing condition and subsequent topoisomerase treatment, such superhelicity was relieved before cruciform extrusion. In this way, we can prepare different topoisomers of the same plasmid, both cruciform-extruded DNA and not extruded DNA. We tested the effect of the cruciform on the translocation-like reaction in the cell using a mixture of cruciform and non-cruciform plasmids. The frequency of the translocation-like reaction was found to depend on the proportion of the cruciform-extruded plasmid DNA (Figure 1A; Inagaki et al., 2009). These results suggest that cruciform extrusion at the palindromic region induces PATRR-mediated translocation.

Notably however, in living cells the conversion of a DNA structure from that of standard B DNA to cruciform DNA is unlikely to occur under normal physiological conditions from a point of view of thermodynamics. Cruciform extrusion at the palindromic region occurs only when the DNA has strong free negative superhelicity. Theoretically, such superhelicity would potentially occur only at the post-meiosis stage in late spermatogenesis. At this developmental stage, histones are replaced by protamines to reduce the cell size (Gaucher et al., 2010). During histone removal, DNA has a transient excess of negative supercoiling, which might induce cruciform extrusion at the palindromic DNA that leads to translocation formation (Boissonneault, 2002). It is highly possible that PATRR-mediated translocations occur at this developmental stage of spermatogenesis (Kurahashi et al., 2010).

Although the post-meiosis hypothesis is captivating, there is some evidence contradicting this hypothesis. One example is the presence of somatic mosaicism of the t(11;22) translocation and normal cells in humans (Kurahashi et al., 2000b). This indicates that the t(11;22) translocation in this case was generated during the mitotic cell cycles after fertilization. Another example is the existence of *de novo* cases of Emanuel syndrome (Kurahashi et al., 2000b). Emanuel syndrome generally occurs via 3:1 segregation of the translocation chromosomes during meiosis I in a t(11;22) balanced carrier. However, a *de novo* Emanuel syndrome case would have arisen via 3:1 segregation of the t(11;22) chromosomes during the pre-meiotic somatic cell cycles of gametogenesis.

## Analysis of the PATRR by next-generation sequencing

In addition to PATRR11 and PATRR22, a dozen PATRRs have been found at other translocation breakpoints. A recurrent t(17;22)(q11.2;q11.2) translocation was found in neurofibromatosis type 1 patients (Kehrer-Sawatzki et al., 2002; Kurahashi et al., 2003). Identification of another recurrent translocation between 8q24.1 and 22q11.2 led to the definition of a new malformation syndrome (Sheridan et al., 2010). Other PATRRs at 4q35.1, 1p21.2, 3p14, and 9p21 were identified at the breakpoints of non-recurrent constitutional translocations (Nimmakayalu et al., 2003; Gotter et al., 2004; Tan et al., 2013; Kato et al., 2014). These PATRRs share little homology but have features of AT-richness and symmetric palindromic structure in common. Intriguingly, all of the palindrome-mediated translocations occur between one PATRR and another PATRR.

We attempted to perform genome-wide screening of *de novo* PATRR-mediated translocations to identify unknown PATRRs using next-generation sequencing. We used the PATRR22 sequence as bait for the detection of any unknown sequences next to the PATRR22 due to *de novo* translocation. However, several difficulties were encountered. We could not confirm the presence of the translocation because most of the PATRR-mediated non-recurrent translocations occurred at a frequency below the detection levels of PCR using sperm from normal healthy donors. Furthermore, we could not analyze the novel translocation junction because the partner sequence could not be mapped to the human reference sequence. None of the translocation-related PATRR sequences identified to date appear in the human genome assembly.

Although the genome projects for many organisms including humans determined their complete nucleotide sequences, difficult-to-sequence regions remain as “gaps.” Recent novel sequencing technologies have made it possible to access some of the gaps and provide more precise genomic data (Chaisson et al., 2015). The PATRR sequences do not appear even in such human reference databases. Palindrome sequences are one such type of a difficult-to-sequence region due to a “triple whammy” of factors affecting sequence analysis: the palindromic sequences are generally refractory to cloning to vectors, PCR amplification, and Sanger sequencing (Inagaki et al., 2005; Lewis et al., 2005). These features are due to the nature of the palindromic sequence itself. The longer the palindrome, the more difficult its analysis.

## Deep sequencing of the PATRR region has generated a novel hypothesis

We applied next-generation sequencing technology to determine the complete sequence of the PATRR on 8q24, which was found at the breakpoint of t(8;22)(q24;q11) (Mishra et al., 2014). Sequencing of a random sheared library of PCR products and reconstruction of the original DNA via the computer-aided alignment of thousands of DNA molecules allowed us to successfully determine the entire PATRR8 (Figure 1B). The next-generation sequencing method does not require cloning and can directly analyze numerous DNA molecules at the same time. Although this strategy still requires PCR to amplify the single molecules and improve signal detection, the random digestion of the palindrome increases the chance of generating asymmetric cleavages of the palindromic center, which improves the PCR efficiency.

By means of this system, we determined the entire PATRR8 sequence, even at the center of the symmetry. This PATRR8 sequence allowed us to develop t(8;22)-specific PCR primers to analyze the junction fragments. The breakage always occurred at the center of the PATRR8 and PATRR22. The fusion was accompanied by the deletion of small nucleotides at the breakpoint regions. Interestingly, the nucleotide sequences around the junctions are identical between the der(8) and der(22) (Mishra et al., 2014). This cannot happen if the two breakages at the PATRR8 and PATRR22 occur independently and are followed by random nucleotide deletion at the breakage ends. This implies coordinated processing of PATRR8 and PATRR22. Similar features of identical junctions in the two derivative chromosomes were also found in t(11;22) and t(17;22) (Kurahashi and Emanuel, 2001a; Kurahashi et al., 2003).

The standard models for gross chromosomal rearrangement include the breakage-first model and the contact-first model (Misteli and Soutoglou, 2009). In the breakage-first model, two DNA breaks located far from each other in the nucleus seek each other out to form a fusion chromosome. The artificial translocation model for the observation of the spatiotemporal chromosomal location in living cells revealed the dynamic movement of chromosomes after their breakage (Roukos et al., 2013). On the other hand, according to the contact-first model, translocation takes place between two closely located sites in the nucleus. Our previous data suggested that PATRR11 and PATRR22 are closer than other control chromosomal regions, indicating that this shorter distance might partly contribute to the recurrent nature of the t(11;22) translocation (Ashley et al., 2006). However, these two models do not explain specific translocations between two PATRRs.

**Figure 2:**
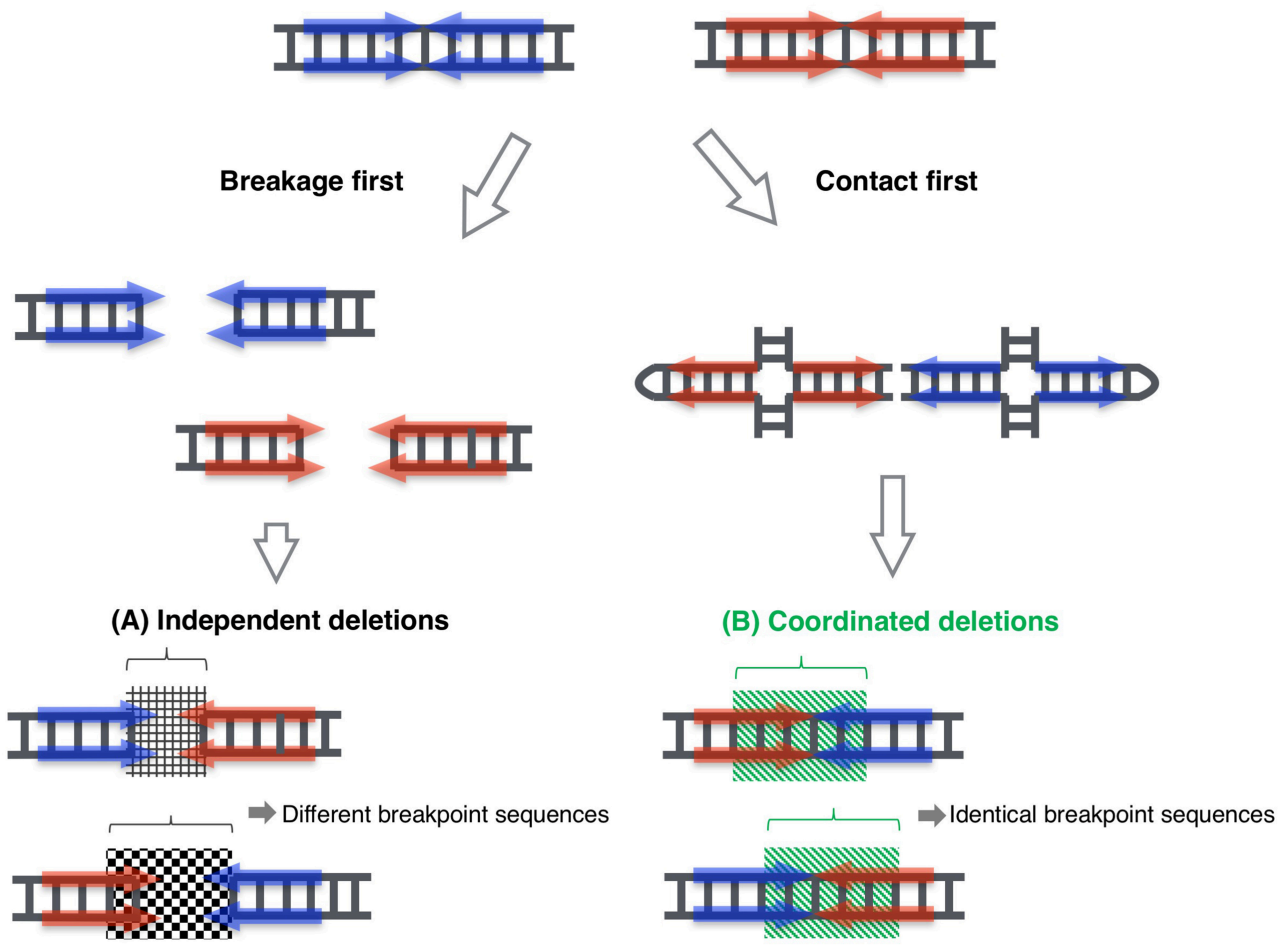
**Proposed model for coordinated joining of derivative chromosomes**. Two derivative chromosomes have an increased likelihood of having identical junction sequences, indicating that exactly equal-sized deletions occurred in each palindrome center, which then joined to form two junction fragments. This phenomenon cannot be explained by the classical model, where the two double-stranded breakages are processed independently **(A)**. This could happen when the breakpoints of the derivative chromosomes are generated in a coordinated manner **(B)**. (Inagaki et al., 2013; Mishra et al., 2014).

Again, the identical sequences of the two derivative chromosomes imply that the two DNA breakage sites are unlikely to have been processed independently. The two derivative chromosomes were likely to be generated in a coordinated manner. Taken together, in the case of a PATRR-mediate translocation, PATRR appears to extrude cruciform structures at some stage during spermatogenesis. The two cruciform DNA molecules seek each other out and finally join together (Figure 2). In our translocation model system in cultured cells described above, the data suggested that two cleavage processes—cleaved diagonal cleavage of the cruciform structure and cleavage of the tip of the hairpin structure—are involved in translocation development (Inagaki et al., 2013). Our data also suggest that the pathway involves the participation of Artemis and ligase IV, which are components of the V(D)J recombination system that act by bringing two chromosomal sites close together and connecting them. In V(D)J recombination, RAG1 and RAG2 proteins bind the two cleavage sites to hold the resulting ends, both of which are specific for the V(D)J recombination machinery in lymphocytes. Similar mechanism is known in a DNA repair system of non-homologous end joining, in which Ku70/80 holds the two broken end until the subsequent repair machinery associate to process and join the ends (Deriano and Roth, 2013). Artemis and ligase IV as well as DNA-PK and other factors also participate in the joining reactions. It is possible that a part of such systems, or other novel factors might be involved in the contact between the two extruded cruciform structures and in keeping them in position during processing until the two derivative chromosomes are generated. We are now investigating how two cruciform DNA molecules come close together to elucidate the third mechanistic model that leads to recurrent chromosomal translocations in humans. Such investigation of dynamics of the cruciforms in nuclei will shed light on the role of non-B DNAs in gross chromosomal rearrangements in other eukaryotes.

## Author contributions

HI and HK wrote the initial manuscript. All authors discussed the text and commented on the manuscript.

## Funding

This study was supported by Grants-in-Aid for Scientific Research (HI, HK) and the MEXT-Supported Program for the Strategic Research Foundation at Private Universities (HK) from the Ministry of Education, Culture, Sports, Science, and Technology of Japan, and a Health and Labour Sciences Research Grant (HK) from the ministry of Health, Labour and Welfare of Japan.

### Conflict of interest statement

The authors declare that the research was conducted in the absence of any commercial or financial relationships that could be construed as a potential conflict of interest.

## Abbreviations

PATRR: palindromic AT-rich repeat.
